# LV geometric and substrate remodelling in patient with Ebstein anomaly - a deep insight from MRI T1 mapping fibrosis imaging

**DOI:** 10.1186/1532-429X-18-S1-P159

**Published:** 2016-01-27

**Authors:** Dan Yang, Jiayu Sun, Ke Wan, Yong Luo, Hong Liu, Wei Cheng, Tianjiang Zhang, Yucheng Chen

**Affiliations:** 1 grid.412901.f0000000417701022Cardiology Division, West China Hospita Sichuan University, Chengdu, China; 2 grid.13291.380000000108071581Radiology Department, West China Hospital, Sichuan University, Chengdu, China; 3 Siemens MR Northeastern Collaboration, Beijing, China

## Background

Ebstein anomaly is a kind of congenital defect of tricuspid valve mainly affecting the right ventricle, recent evidence have suggested the LV geometry remodeling in patient with Ebstein anomaly due to potential RV-LV coupling. The aim of this study-we investigated the interaction between abnormal right ventricle and left ventricle and whether myocardial fibrosis is the basis of abnormal left ventricle remodeling in ebstein abnomaly by using T1 mapping technique of cardiac magnetic resonance.

## Methods

Twenty patients with ebstein's anomaly and twenty matched normal controls were prospectively included in this study. All subjects received a comprehensive cardiac MRI scanning according to a standard protocol, which consisted of pre- and post-contrast T1 mapping for calculating ECV to quantify myocardial fibrosis of left ventricle. LV geometry and function data. Bi-ventricular volume, function and severity of ebstein anomaly were also analyzed.

## Results

In patients with Ebstein anomaly, LV volume was mildly decreased and EF also mildly reduced .ECV of LV myocardium in ebstein was larger than that of normal controls (28.76 ± 4.40 vs 23.39 ± 3.56 p = 0.003). ECV correlated well with severity index of RV malformation and LVEF in Ebstein's patients group. The native-T1 value of LV myocardium in ebstein's was longer than that of in normal controls with p = 0.04.

## Conclusions

LV myocardial fibrosis and remodeling evidently existed in patients with ebstein's anomaly which was strongly correlated to the severity index of ebstein. Myocardial fibrosis plays a role in LV remodeling which directly results from LV deformity in ebstein' patients.

**Figure 1:**
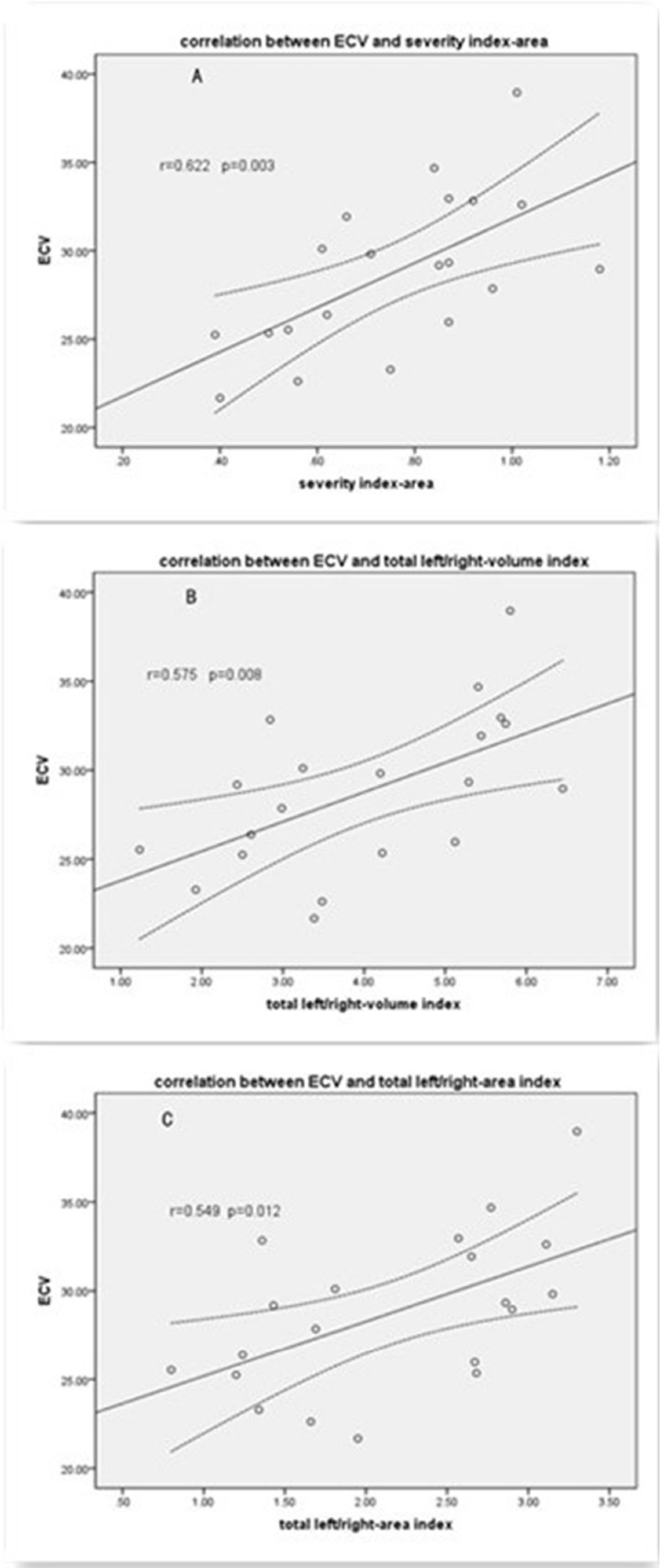
**Three methods were used to define the severity of RV malformation listed as severity index-area (A), total left/right- volume index (B) and total left/right -area index (C),severity index-area is the ratio of area of right atrium and atrialized RV to area of functional RV and left heart ,total left/right-volume index is the ratio of volume of total left heart to volume of right heart**. Total left/right-area index is the ratio of area of total left heart to area of right heart. And the three method were measured on the 4 chamber at the end-diastole phase.

